# Macrophage mannose receptor, CD206, predict prognosis in patients with pulmonary tuberculosis

**DOI:** 10.1038/s41598-018-31565-5

**Published:** 2018-09-03

**Authors:** Yuzo Suzuki, Masahiro Shirai, Kazuhiro Asada, Hideki Yasui, Masato Karayama, Hironao Hozumi, Kazuki Furuhashi, Noriyuki Enomoto, Tomoyuki Fujisawa, Yutaro Nakamura, Naoki Inui, Toshihiro Shirai, Hiroshi Hayakawa, Takafumi Suda

**Affiliations:** 1 Second Division, Department of Internal Medicine, Hamamatsu University School of Medicine, Hamamatsu, Japan; 2 Respiratory Medicine, Tenryu Hospital, National Hospital Organization, Hamamatsu, Japan; 3 Respiratory Medicine, Shizuoka General Hospital, Shizuoka, Japan

## Abstract

Tuberculosis (TB) remains a leading cause of fatal infectious disease. Accumulations of macrophages are found in infected sites; thus, we hypothesized that a marker of activated macrophages may be related to prognosis of pulmonary TB (PTB). This study investigated serum soluble macrophage mannose receptor, sCD206, in PTB and examined its clinical significance. First, the concentration of sCD206 was measured in the sera of 96 patients with PTB (Tenryu cohort), and in pleural effusions from 29 patients with TB pleurisy. These were verified in another independent cohort (Shizuoka cohort). We found increased concentrations of sCD206 in sera, but not in pleural effusions of PTB patients. Notably, PTB patients with poor prognosis showed significantly higher levels of serum sCD206. At a cut-off value of 1,600 ng/mL in the Tenryu cohort, sCD206 predicted prognosis of PTB with area under the curve 0.847, sensitivity 77.3%, and specificity 86.5%. These results were validated in the Shizuoka cohort. Pathological analyses showed concordance of enhanced CD206 expression in lung and pleural tissues with caseating granuloma in TB. Serum sCD206 increased in PTB and was associated with prognosis. sCD206 is a potential biomarker for PTB.

## Introduction

Tuberculosis (TB) remains a worldwide health problem with 10.4 million new cases each year, and more than 1.7 million deaths in 2016^1^. These incidences have gradually declined, but have not achieved the goal of the World Health Organization (WHO) End TB Strategy target^2,3^. For first-line defence against *Mycobacterium tuberculosis* (*Mtb*), macrophages are an essential component for the complex immune reactions required against this pathogen. Tissue-resident alveolar macrophages are the initial defence against Mtb in the lung. Upon infection, blood monocyte- derived macrophages are recruited to the site of infection where they provide the innate defence, in addition to initiating and controlling the adaptive immune response^4–6^.

CD206, known as mannose receptor and C-type lectin, is primary expressed on the surface of macrophages and immature dendritic cells, where it acts as a pattern recognition receptor (PRR)^7–10^. CD206 interacts with glycoproteins and glycolipids found on the surface of pathogens, including viruses, fungi, and bacteria (e.g., *Mtb*); thus, CD206 plays a role in immune recognition of pathogens, following antigen internalization and presentation^8–10^. Additionally, CD206 functions in clearance of glycoproteins from circulation, including sulphated glycoprotein hormones and glycoproteins released in response to pathological events^11^. Following proteolytic cleavage from macrophage membranes, soluble forms of CD206 (sCD206) are found in the periphery. Similar to membrane forms, soluble forms of CD206 also recognize sulphated and mannosylated carbohydrates, and can alter the innate and adaptive immune responses^12,13^.

CD206 is a marker of alternatively activated macrophages, known as M2 macrophages. In contrast to classically activated macrophages (known as M1 macrophages), M2 macrophages are anti-inflammatory and contribute to tissue repair, resolution of inflammation, induction of immune tolerance, and protection from excessive inflammation^8,9,14^. Interestingly, the ability of CD206 to aid pathogen internalization is exploited by *Mtb* to facilitate infection within macrophages^15^, suggesting CD206 can serve as an immune escape mechanism and as a novel therapeutic target. Therefore, we hypothesized that CD206 may provide a potential biomarker in TB, where macrophages exhibit an immune regulatory phenotype. The present study evaluated serum and pleural CD206 concentrations in patients with pulmonary TB (PTB), and examined membrane-bound CD206 expression in the lung and pleural tissues by using immunohistochemistry.

## Results

### Clinical characteristics

Clinical characteristics of PTB patients are summarized in Table 1 and Supplement Table 1. Proportions of age, gender, and mortality rate did not differ between the cohorts. Most patients demonstrated lean body weight and had lower body-mass index (BMI). TB pleurisy was frequently found in the Tenryu cohort, while cavity lesions in chest radiographs were common in the Shizuoka cohort. There were no significant differences in comorbidity, presence of respiratory failure (SaO_2_ < 90%), or impaired consciousness between the two cohorts. Multidrug resistant TB (MDR-TB) was isolated in three patients, and no patients were infected with human immunodeficiency virus (HIV).

**Table 1:** Clinical characteristics of patients with pulmonary tuberculosis.

	Tenryu Cohort (n = 96)	Shizuoka Cohort (n = 112)	p-value
Sex, M/F	62/34	71/41	0.886
Age, yr	72.4 ± 21.5*	71.8 ± 19.6	0.410
Body mass index	19.1 ± 3.4 (n = 78)	18.8 ± 3.4 (n = 110)	0.347
Mortality	22 (22.9%)	26 (23.2%)	1.000
Current smoker	14 (14.6%)	17 (15.2%)	1.000
**System involved**			
Pulmonary tuberculosis only	53 (55.2%)	81 (72.3%)	0.013
Tuberculous pleurisy	37 (38.9%)	25 (22.3%)	0.010
Disseminated tuberculosis	12 (12.5%)	9 (8.0%)	0.376
Osteoarticular tuberculosis	3 (3.1%)	4 (3.6%)	1.000
Bronchial tuberculosis	0 (0%)	4 (3.6%)	0.126
Tuberculous colitis	2 (2.1%)	1 (0.9%)	1.000
**Radiographic findings**			
Cavity	26 (27.1%)	63 (56.3%)	<0.001
**Microbiological findings**			
Suputum smear (0, 1+, 2+, 3+, 4+)	8, 47, 15, 16, 10	2, 44, 21, 36, 9	0.024
MDR-TB, no. (%)	1 (1.0%)	2 (1.8%)	1.000
**Comorbidity, no. (%)**			
Congestive heart failure	14 (14.6%)	20 (17.9%)	0.576
Chronic pulmonary disease	13 (13.5%)	20 (17.9%)	0.450
Renal disease	6 (6.3%)	4 (3.6%)	0.518
Diabetes mellitus	13 (13.5%)	20 (17.9%)	0.450
Liver disease	3 (3.1%)	3 (2.7%)	1.000
Cerebrovascular disease	22 (22.9%)	20 (17.9%)	0.390
Neoplasm	9 (9.4%)	9 (8.0%)	0.807
Chronic corticosteroid treatment	7 (7.3%)	5 (4.4%)	0.553
**Clinical characteristics on admission**			
Body temperature, °C	36.9 ± 0.7	36.8 ± 0.9	0.509
Heart rate, rate/min	83.7 ± 15.5	86.0 ± 15.8	0.145
Respiratory failure (SaO_2_ < 90%), no. (%)	22 (10.6%)	17 (8.2%)	0.160
Impaired consciousness, no. (%)	5 (5.2%)	4 (3.6%)	0.736
**Laboratory findings**			
BUN, (8.6–21.6 mg/dl)**	19.1 ± 13.1	17.6 ± 10.4	0.665
TP, (6.7–8.1 g/dl)	6.8 ± 1.0	7.1 ± 1.0	0.042
Alb, (3.9–4.9 mg/dl)	3.1 ± 0.8	3.0 ± 0.8	0.283
Cre, (0.70–1.17 mg/dl)	0.80 ± 0.45	0.81 ± 0.39	0.228
WBC, (3600–9200/mm^3^)	6986 ± 2867	7958 ± 3759	0.020
ESR, (2–10 mm/H)	64.7 ± 33.7 (n = 84)	44.5 ± 29.8 (n = 111)	<0.001
CRP, (≦0.10 mg/dl)	5.2 ± 4.7	5.7 ± 6.1	0.914
SAA, (≦10 μg/ml)	380 ± 401 (n = 84)	408 ± 610 (n = 111)	0.474

MDR-TB, multidrug resistant tuberculosis; SaO_2_, arterial oxygen saturation; BUN. blood urea nitrogen; TP, total protein; Alb, alubumin; Cre, creatinine; WBC, white blood cell count**;** ESR, erythrocyte sedimentation rate; CRP, C-reactive protein; SAA, serum amyloid A; PCT, procalcitonin. ^*^Mean ± SD; ^**^Normal range.

Of the PTB patients, non-surviving patients showed older age and lower BMI. Hypoxia and impaired consciousness were more frequently found in non-survivors in both cohorts. Congestive heart failure and neoplastic disease were comorbidities commonly found in non-surviving patients. Laboratory examination revealed that non-surviving patients had malnutrition status, reduced total protein (TP) and albumin (Alb) levels, and elevated inflammatory markers, such as C-reactive protein (CRP) and serum amyloid A (SAA), in the peripheral blood (Supplement Table 1).

### Serum concentrations of soluble CD206 in the Tenryu Cohort

**Figure 1:**
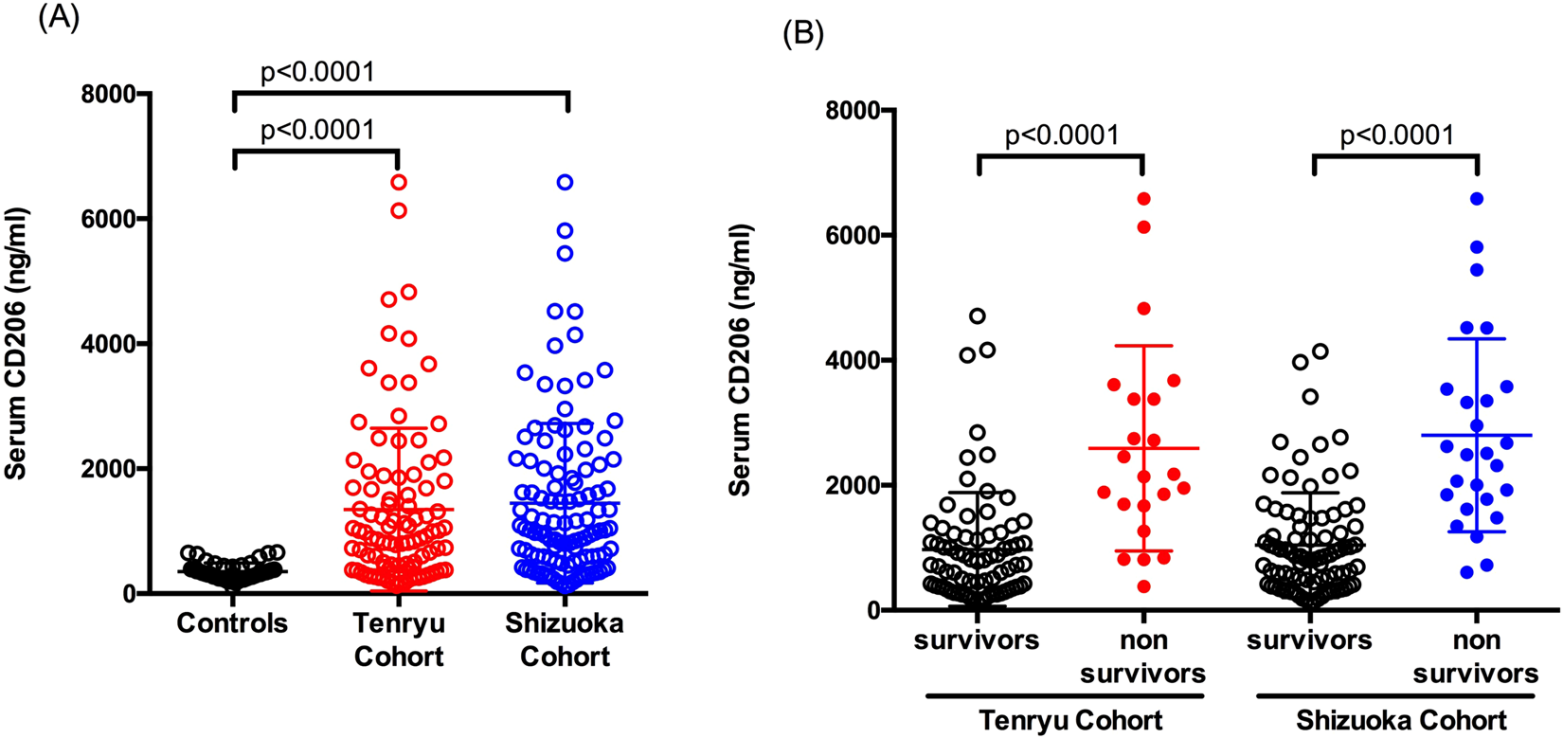
Serum concentrations of sCD206 in patients with pulmonary tuberculosis (PTB) and control subjects (**A**), in PTB patients with non-survivors and survivors (**B**). P values were determined by the Mann-Whitney U test, and the Kruskal-Wallis test followed by Dunn’s multiple comparison test.

Serum concentrations of sCD206 in PTB patients in the Tenryu cohort are presented in Fig. 1. Serum concentrations of sCD206 in PTB patients were more than three times higher than in controls (1,344 ± 1,303 ng/mL vs. 354 ± 138 ng/mL, p < 0.0001; Fig. 1A). Notably, non-surviving PTB patients showed significantly higher serum sCD206 levels than surviving PTB patients (2,590 ± 1,640 ng/mL vs 974 ± 910 ng/mL, p < 0.0001; Fig. 1B). Correlation analyses showed that the serum concentration of sCD206 in PTB was strongly associated with total protein, albumin, lactate dehydrogenase, and inflammatory markers (e.g., CRP and SAA) (Supplement Table 2).

### Serum concentrations of soluble CD206 in the Shizuoka cohort

We separately evaluated serum sCD206 levels in another PTB cohort. Levels of serum sCD206 in PTB patients in the Shizuoka cohort (1,449 ± 1276 ng/mL) were significantly higher than in controls (p < 0.0001) and comparable with levels in the Tenryu cohort (Fig. 1A). Additionally, non-surviving patients showed significantly increased serum sCD206, compared with surviving PTB patients (2,800 ± 1,542 ng/mL vs. 1,041 ± 838 ng/mL, p < 0.0001; Fig. 1B). Correlation analyses are shown in Supplement Table 2, and demonstrated similarity between Shizuoka and Tenryu cohorts.

### Concentration of sCD206 in pleural effusions

**Figure 2:**
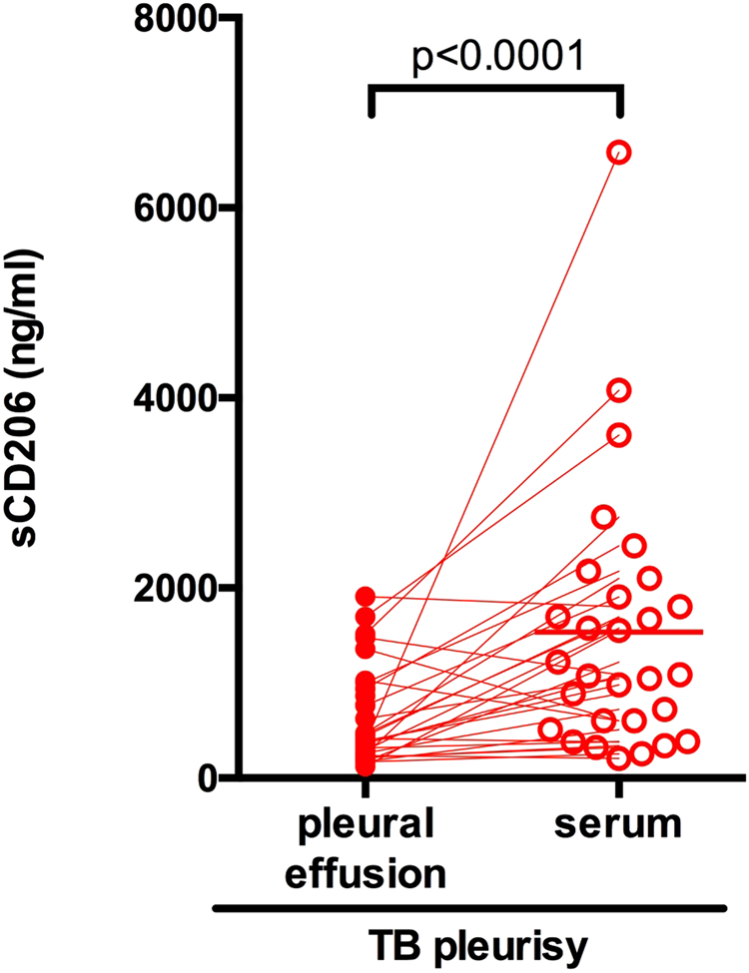
Serum and pleural concentrations of sCD206 in patients with tuberculous (TB) pleurisy. Unfilled and filled circle represent pleural fluids and serum, respectively. P values were determined by the Wilcoxon matched-pairs signed rank test.

Because higher concentrations of sCD206 were found in patients with TB, we hypothesized that pleural sCD206 might be elevated, and subsequently might aid in differential diagnosis of pleural disease. We next evaluated sCD206 levels in pleural effusions in patients with TB pleurisy. Among the 37 Tb pleurisy cases in the Tenryu cohort, plural effusions and sera were simultaneously collected from 29 cases (Supplement Table 3). Compared with serum sCD206 concentration, pleural sCD206 levels were significantly reduced (623 ± 517 ng/mL vs 1,537 ± 1381 ng/mL, p < 0.0001; Fig. 2).

### Prognostic value of sCD206 in patients with PTB

**Figure 3:**
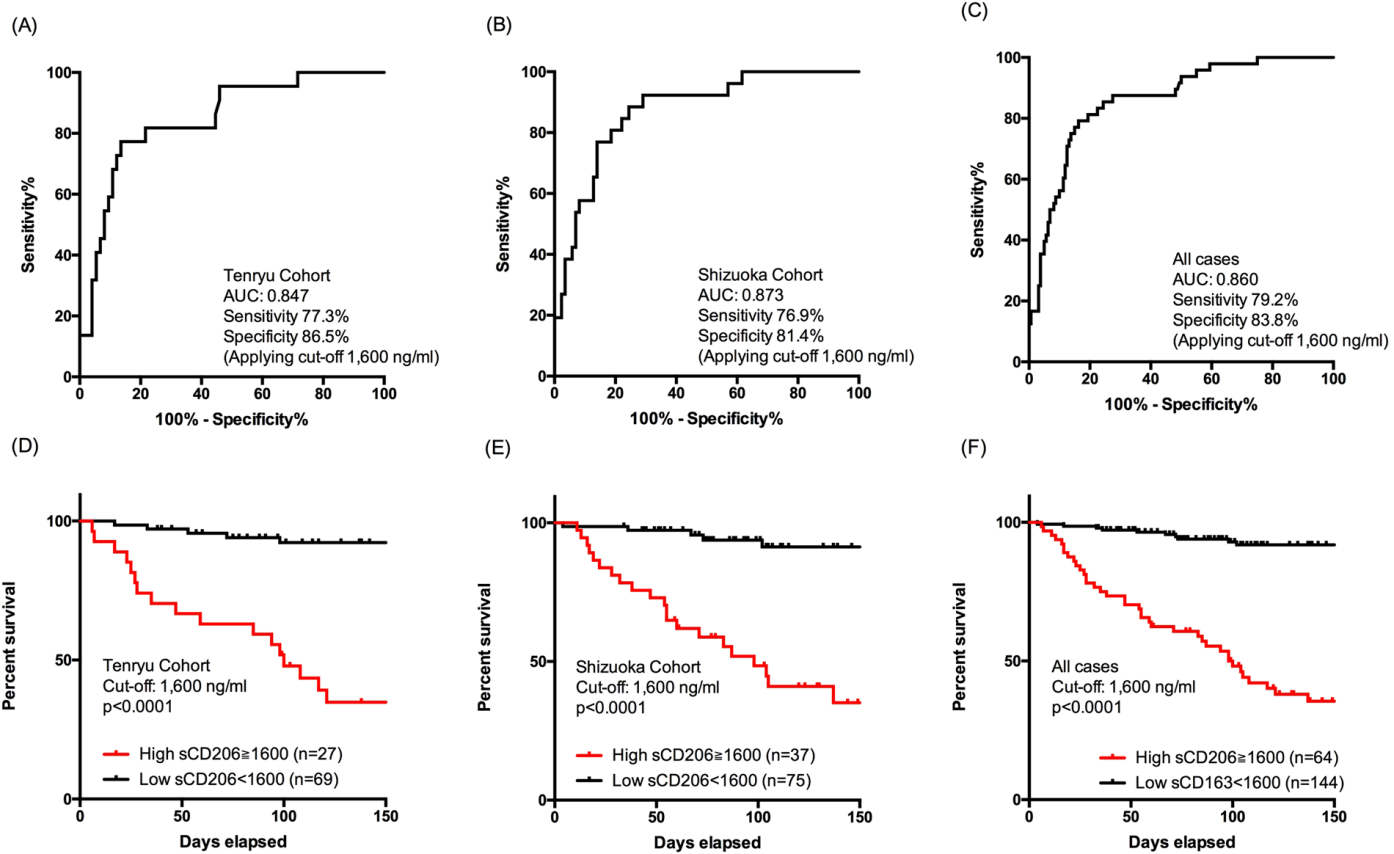
Receiver operator curve analysis of serum concentrations of sCD206 predicting mortality in patients with Tenryu, Shizuoka, and combined cohorts (**A**–**C**). Kaplan-Meier curves of patients with Tenryu, Shizuoka, and combined cohorts according to serum sCD206. P values were determined by the log rank test (**D**–**F**).

To evaluate the potential value of serum sCD206 for predicting the prognosis of PTB, receiver operating characteristic (ROC) analyses were performed by using each cohort separately and combined. The area under curve (AUC) for sCD206 in the Tenryu cohort was 0.847 for predicting mortality (95% confidence interval (CI), 0.756–0.939). With an optimal cut-off value of 1,600 ng/ml, the sensitivity was 77.3% and specificity was 86.5% (Fig. 3A). The AUC in the Shizuoka cohort was 0.873 (95% CI: 0.800–0.946); the sensitivity was 76.9% and specificity was 81.4% by using cut-off values obtained from the Tenryu cohort (1,600 ng/ml, Fig. 3B). Similarly, the AUC in the combined PTB patients was 0.86 (95% CI: 0.801–0.918). The sensitivity and specificity was 79.2% and 83.8%, respectively, when applying the cut-off of 1,600 ng/ml (Fig. 3C). The sensitivities and specificities for predicting mortality with several additional cut-offs are shown in Supplement Table 4. The AUCs of CRP and SAA for predicting prognosis are shown in Supplement Table 5.

We then assessed the prognosis of patients with PTB on either side of this cut-off value by using the Kaplan-Meier method and log-rank test. Groups with high sCD206 levels showed significantly higher mortality rates than groups with low sCD206 levels (Tenryu cohort: 63.0% vs. 7.2%, p < 0.0001; Shizuoka cohort: 52.3% vs. 4.4% p < 0.0001; combined cohort data: 59.4% vs. 6.9%, p < 0.0001; Fig. 3D–F).

Finally, to examine the prognostic values of sCD206 with regard to the outcomes among all PTB cases, we performed Cox proportional hazards analyses (Table 2). Univariate analyses showed several covariates, including sCD206, were statistically significant. Subsequently, clinically important covariates, including age, congestive heart failure, and respiratory failure, were used for multivariate analyses. Because of potential confounders or statistical limitations, several covariates were excluded. As shown, sCD206 was significantly associated with the outcome of PTB. In contrast, pleural sCD206 was not associated with prognosis of TB pleurisy (HR 0.986, 95% CI: 0.999–1.001; p = 0.986).

**Table 2:** Prediction of mortality with pulmonary tuberculous patients: univariate and multivariate analyses.

Predictor	HR	95% CI	p-value	HR	95% CI	p-value
	Univariate analysis			Multivariate analysis		
Age, yr	1.052	1.025–1.080	<0.001	1.033	1.001–1.067	0.043
Sex, male	0.685	0.387–1.212	0.194			
Body mass index, Kg/m^2^	0.829	0.749–0.919	<0.001			
Current smoker	0.332	0.103–1.068	0.064			
Pulmonary tuberculosis only	0.615	0.348–1.085	0.093			
Disseminated tuberculosis	2.139	1.036–4.418	0.040			
TB pleurity	1.681	0.942–2.998	0.079			
Congestive heart failure	3.178	1.740–5.803	<0.001	1.559	0.812–2.996	0.182
Neoplasm	3.052	1.519–6.131	0.002			
Respiratory failure (SaO_2_ < 90%)	5.119	2.900–9.036	<0.001	1.040	0.969–1.115	0.277
Impaired consciousness	8.099	3.701–17.720	<0.001			
BUN, /mg/dl	1.027	1.009–1.045	0.004			
TP, mg/dl	0.392	0.299–0.515	<0.001			
Alb, /mg/dl	0.278	0.182–0.424	<0.001	0.559	0.317–0.987	0.045
CRP, /mg/dl	1.102	1.066–1.140	<0.001	1.057	1.005–1.111	0.031
CD206, /100 ng/ml	1.050	1.036–1.064	<0.001	1.025	1.006–1.044	0.009

HR; hazard ratio, CI; confidence interval, BUN; blood urea nitrogen, TP, total protein, Alb; alubumin, CRP; C-reactive protein, SAA, serum amyloid A.

### Expression of CD206 in the lung and pleural tissues of patients with PTB

**Figure 4:**
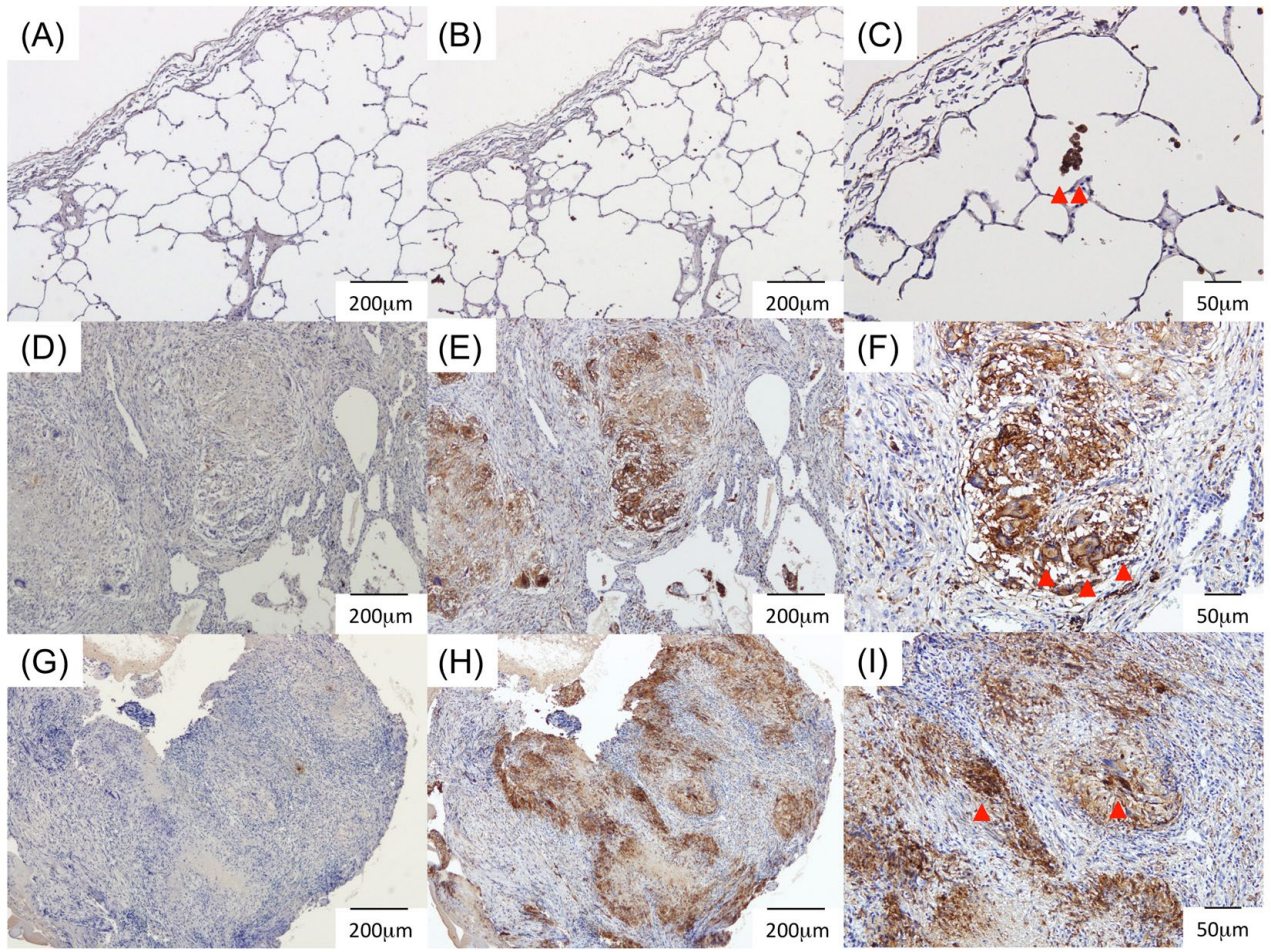
Immunohistochemical staining. Lung sections from an early lung cancer patient (**A**–**C**), patient with tuberculosis (TB, **D**–**F**), and pleural tissues from patient with TB pleurisy (**G**–**I**). CD206 positive macrophages were observed (arrow head in **C**). CD206 were stained with the caseating granulomas (**E**,**H**), and multinucleated giant cells (arrow head in **F**,**I**). (**A**,**D**,**G**) Isotype controls (X40). (**B**,**E**,**H**) Anti-CD206 (X40). **(C**,**F**,**I**) Anti-CD206 (X100).

To determine the source of sCD206 in the sera and pleural effusions of patients with TB, we assessed CD206 expression in lung and pleural specimens from patients with PTB and TB pleurisy by using immunohistochemistry (Fig. 4). The lung sections from an early lung cancer patient were also examined as a control. In normal lung sections, CD206 expression was found with alveolar macrophages (Fig. 4C). Conversely, CD206 staining was observed in epithelioid granulomas and multinucleated giant cells in both lungs and pleural tissues from PTB and TB pleurisy patients (Fig. 4F,I).

## Discussion

The present study evaluated the clinical significance of the alternative macrophage marker CD206 in patients with PTB by using two independent cohorts. PTB patients had elevated levels of serum sCD206, compared with controls; enhanced expression of CD206 was also found in epithelial granulomas in the lung and pleural tissues from PTB patients. Compared with pleural effusions, serum sCD206 levels were significantly elevated, which was associated with poor outcome in both cohorts. Particularly, more than eight times higher mortality was found in PTB patients with elevated sCD206. Collectively, our results suggested that CD206 could predict prognosis of patients with PTB.

Hallmarks of macrophages include diversity and plasticity, as macrophages express an array of PRRs. Among these PRRs, CD206 is preferentially expressed on the surface of M2 macrophages and recognizes various pathogens, including *Mtb*, via its mannosylated surface structure^7,16^. CD206 ligation on non-activated macrophages produces an anti-inflammatory response by stimulating the release of anti-inflammatory cytokines^17^ and inhibiting the production of pro-inflammatory IL-2^18^ and reactive oxygen species^19^. During *Mtb* infection, dynamic changes occur in macrophage polarization; macrophage transformation from M1 to M2 is observed over time following *Mtb* infection^20^, and M2 macrophages are spatially dominant in tissue surrounding the caseum^21^. Indeed, enhanced expression of CD206 was found in lung and pleural tissues from patients with PTB. The membrane form of CD206 on the surface of M2 macrophages undergoes proteolytic cleavage, leading to the release of sCD206^9,10^. Notably, detailed mechanisms for generating sCD206 are not fully known, and direct assessments of sCD206 and CD206-expressing macrophages at the site of infection were not performed in the present study; however, high concentrations of sCD206 might represent activation of M2 macrophages in PTB.

M2 macrophages are involved in the resolution of inflammation and tissue repair, preventing excessive inflammation^8,9,14^; CD206 functions as a scavenger receptor to maintain homeostasis by clearing lung environments^9,10^. However, engagement of *Mtb* with CD206 initiates the phagosomal niche, enhancing the potential for *Mtb* survival in the macrophage^15^. The precise immunological role of elevated sCD206 in the periphery in patients with PTB was not elucidated in the present study. However, our results showed that sCD206 correlated with inflammatory markers and disease severity, providing prognostic value. Collectively, our results indicated involvement of CD206 in the pathogenesis of *Mtb* infection.

The present study was the first to evaluate pleural sCD206 levels in pulmonary disease. Tb pleurisy is considered to be a delayed allergic reaction to mycobacterial antigens^22,23^; thus, *Mtb* is rarely cultured from pleural fluids. Indeed, an increased number of regulatory T cells and a decreased number of effector T cells were found in pleural fluid in patients with TB pleurisy^24,25^. Consistent with these reports, levels of immunoregulatory molecules indoleamine 2,3-dioxygenase and CD163 were elevated in pleural effusions of patients with TB pleurisy, compared with levels in the blood^26,27^; this suggested induction of immune tolerance in the thoracic cavity in patients with TB pleurisy. Thus, we expected elevated pleural sCD206 concentrations in pleural fluids from patients with TB pleurisy. Unexpectedly, pleural sCD206 levels were significantly reduced, compared with levels in blood. Similar to our results, Jiang *et al*. reported a greater number of M1 macrophages and a reduced number of M2 macrophages in pleural fluids from patients with TB pleurisy, compared with levels in the blood^28^. Therefore, these results suggested distinct polarization between macrophages and T cells, as well as complex *Mtb*-immune cell interactions in TB pleurisy.

The prevalence of TB in Japan is gradually declining; it was 13.9 per 100,000 in 2016, which remains higher than in Western countries (typically <10 per 100,000). The incidence of MDR-TB was reported to be 0.5%; more than 70% of patients were over 60 years of age^29^. Thus, most of the subjects in our cohorts were older and exhibited comorbidities, which resulted in a higher mortality rate. Although standard regimens have been established, concerns remain in the treatment of TB, including extended treatment duration, spread of MDR-TB and extensively drug-resistant TB, and HIV co-infection. Therefore, the development of adjunct host-directed therapies (HDTs) by modulating host inflammatory pathways and augmenting components of host innate and adaptive immune mechanisms is needed to improve cure rates in *Mtb* infection^30,31^. Thus, although the present study involved relatively small cohorts, this investigation of prognostic determinants might contribute to the development of HDTs.

There were several limitations in this study. First, as described above, the cohort analysed in the study was relatively small. Second, a wide array of TB biomarkers are available—for diagnosis, assessment of the risk of disease development (latent *Mtb* infection), and measurement of treatment outcomes—with different platforms including cytokines, gene expression, and proteins^32–34^. Among these, TB-specific host biomarkers have been reported; interferon (IFN)-inducible neutrophil-driven transcriptional signature was associated with TB diagnosis and correlated with response to treatment^35^. Third, as with most surrogate markers in TB, sCD206 is not specific for TB and exhibits a demonstrable false-positive rate. Thus, interpretations should be made with awareness of these limitations.

In summary, the present study examined the clinical usefulness of the macrophage-mannose receptor, CD206, as a biomarker in patients with PTB. Enhanced CD206 expression in lung and pleural tissues from PTB patients, and higher concentrations of serum sCD206, were associated with reduced survival. These results warrant further study to determine the clinical usefulness of sCD206 as a biomarker for PTB.

## Methods

### Subjects

This prospective study was conducted by using two cohorts of patients who had presented at referral hospitals for the treatment of TB in Shizuoka, Japan. A cohort of 96 consecutive PTB patients admitted to Tenryu Hospital between January 2010 and December 2011 (Tenryu cohort), and a cohort of 112 consecutive PTB patients hospitalized at Shizuoka General Hospital between March 2010 and February 2011 (Shizuoka cohort), were enrolled in this study. This study also included sera from 42 age- and gender-matched subjects (30 men and 12 women, mean age of 72 years) who visited Hamamatsu University Hospital for health checks, as a control group. None of the control subjects have PTB, non-mycobacterium tuberculosis, or fungal infections, as assessed by chest radiographs.

This study was approved by the ethics committees of Hamamatsu University School of Medicine, Tenryu Hospital, and Shizuoka General Hospital (E15-167), and was carried out in accordance with approved guidelines. Written informed consent was obtained from all subjects in accordance with institutional guidelines. The study was registered in the University Hospital Medical Information Network in Japan (http://www.umin.ac.jp/. UMIN000003400).

### Sample Collection

Blood samples were drawn at the time of admission before beginning treatment with anti-TB drugs. Pleural effusions were simultaneously collected from 29 PTB patients from the Tenryu cohort who showed combined TB pleurisy, with a standard thoracentesis technique, and/or thoracic fiberscopes. Serum and pleural effusions were frozen at −80 °C until analyzed; routine laboratory examinations, such as blood cell counts and biochemical analyses, were subsequently performed. sCD206 levels were determined by using an enzyme-linked immunosorbent assay kit (Ray Biotech, Norcross, GA, USA). SAA levels were measured for 84 patients in the Tenryu cohort and 111 patients in the Shizuoka cohort, respectively, at the physician’s discretion.

### Diagnosis

PTB was diagnosed by isolation of *Mtb*, along with the presence of new radiographic pulmonary infiltration. All sputum samples from PTB patients were confirmed *Mtb*-positive in both culture and polymerase chain reaction assay. Criteria for disseminated TB comprised miliary infiltrates on chest radiographs or computed tomography with numerous 2–3-mm nodules throughout the lung field^36^.

TB pleurisy was diagnosed based on one or more of the following criteria: 1) Isolation of *Mtb* from pleural fluid or tissue; 2) granulomas in pleural tissue that stained positive for acid-fast bacilli; 3) granulomas in pleural tissue that stained negative for acid-fast bacilli, but showed a response to anti-TB treatment; and/or 4) a TB-positive sputum culture. Pleural tissues were obtained by percutaneous techniques and thoracoscopic pleural biopsy.

### Immunohistochemistry

Lung and pleural specimens were obtained from one patient with early lung cancer and PTB by partial resection and three patients with TB pleurisy by using thoracoscopy under local anaesthesia. Tissues were fixed in 10% formalin and embedded in paraffin. Deparaffinized sections (5-μm thick) were immersed in epitope retrieval solution (Target Retrieval Solution S1700; Dako North America, Inc., Carpinteria, CA, USA) and preheated at 120 °C for 10 min. After blocking endogenous peroxidase activity with 3% H_2_O_2_ for 15 min, slides were incubated overnight with a mouse anti-human CD206 monoclonal antibody (15 µg/ml; R&D Systems, Minneapolis, MN, USA) or IgG2b at 4 °C. Subsequently, sections were incubated with visualization reagent (ChemMate Envision kit; Dako Japan, Inc., Tokyo, Japan) for 30 min, followed by counterstaining with haematoxylin.

### Statistical analyses

Discrete variables are expressed as counts (percentage), and continuous variables are expressed as the mean ± SD, unless otherwise specified. The Mann-Whitney test and the Wilcoxon matched-pairs signed rank test were used for continuous variables. Categorical data were compared between groups by using Fisher’s exact test for independence. Correlations between sCD206 and clinical parameters were analysed by using Spearman’s rank correlation technique. Overall survival time was measured from date of PTB diagnosis. To examine the ability of sCD206 to predict mortality in patients with PTB, two distinct cohorts (Tenryu cohort as discovery cohort and Shizuoka cohort as validation cohort) were evaluated. An ROC curve was used to evaluate the ability of sCD206 to discriminate between patients who survived and who died during admission. The optimal cut-off value of sCD206 in the Tenryu cohort, which ensured the best combinations of sensitivity and specificity, was obtained. The optimal cutoff value in the Tenryu cohort was applied to evaluate the Shizuoka cohort and combined cohort data. Cumulative survival probabilities were estimated by using the Kaplan-Meier method with cut-off vales obtained from Tenryu cohort. The log-rank test was used to compare survival among patients. Univariate and multivariate analyses were performed with Cox proportional hazards regression analysis with combined cohort subjects to predict mortality. Among the statistically significant covariates in the univariate analyses, several covariates were excluded because of potential confounders and statistical limitations. Statistical analyses were performed by using GraphPad Prism Version 6 (GraphPad Software, San Diego, CA, USA) and SPSS Statistics (IBM Corporation, Armonk, NY, USA) software. All analyses were two-tailed and P values < 0.05 were considered significant.

## Electronic supplementary material

Supplement Table

## Data Availability

The data are available from the authors upon reasonable request.
