# Supplementary material for: Macrophage mannose receptor, CD206, predict prognosis in patients with pulmonary tuberculosis
Source: Sci Rep. 2018 Sep 3;8:13129. doi: 10.1038/s41598-018-31565-5 (PMC6120933; doi:10.1038/s41598-018-31565-5)
Supplement: Supplementary file 1 — Supplement Table [file 41598_2018_31565_MOESM1_ESM.pdf]

Macrophage mannose receptor, CD206, predict prognosis in patients with pulmonary tuberculosis

Yuzo Suzuki, M.D., Ph.D.[1], Masahiro Shirai, M.D., Ph.D.[2], Kazuhiro Asada, M.D., Ph.D.[3], Hideki Yasui, M.D., Ph.D. [1], Masato Karayama, M.D., Ph.D. [1], Hironao Hozumi, M.D., Ph.D. [1], Kazuki Furuhashi, M.D., Ph.D. [1], Noriyuki Enomoto, M.D., Ph.D. [1], Tomoyuki Fujisawa, M.D., Ph.D. [1], Yutaro Nakamura, M.D., Ph.D. [1], Naoki Inui, M.D., Ph.D.[1], Toshihiro Shirai, M.D., Ph.D.[3], Hiroshi Hayakawa, M.D., Ph.D.[2], Takafumi Suda, M.D., Ph.D[1]

[1]Second Division, Department of Internal Medicine, Hamamatsu University School of Medicine, Hamamatsu, Japan

[2] Respiratory Medicine, Tenryu Hospital, National Hospital Organization, Hamamatsu, Japan

[3] Respiratory Medicine, Shizuoka General Hospital, Shizuoka, Japan

Corresponding author: Yuzo Suzuki, M.D., Ph.D.

12 ***Supplemental Table 1. Clinical characteristics of patients with pulmonary tuberculusis according to the mortality***

	Tenryu Cohort (n=96)			Shizuoka Cohort (n=112)		
	suvivors (n=74)	non-survivors (n=22)	*p*-value	suvivors (n=86)	non-survivors (n=26)	*p*-value
Sex, M/F	50 / 24	12 / 10	0.313	56 / 30	15 / 11	0.496
Age, yr	69.2±23.2	83.1±8.2	0.034	68.5±20.0*	82.5±13.6	<0.001
Body mass index	19.6±3.2	17.2±3.6	0.021	19.3±3.4	17.3±2.9	0.043
Current smoker	1 (4.6%)	13 (17.6%)	0.178	15 (17.4%)	2 (7.7%)	0.351
**System involved**						
Pulmonary tuberculosis only	43 (58.1%)	10 (45.5%)	0.335	65 (75.6%)	16 (61.5%)	0.211
Tuberculous pleurisy	27 (36.5%)	10 (45.5%)	0.465	16 (18.6%)	9 (34.6%)	0.108
Disseminated tuberculosis	7 (9.5%)	5 (22.7%)	0.138	5 (5.8%)	4 (15.4%)	0.209
Osteoarticular tuberculosis	2 (2.9%)	1 (3.9%)	1.000	3 (3.5%)	1 (3.9%)	1.000
Bronchial tuberculosis	0 (0%)	0 (0%)	-	4 (4.7%)	0 (0%)	0.572
Tuberculous colitis	2 (2.9%)	0 (0%)	1.000	0 (0%)	1 (3.9%)	0.232
**Radiographic findings**						
Cavity	14 (18.9%)	12 (54.6%)	0.002	51 (59.3%)	12 (46.2%)	0.265
**Microbiological findings**						
Suputum smear (0, 1+, 2+, 3+, 4+)	7, 37, 14, 13, 3	1, 10, 1, 3, 7	0.004	1, 32, 18, 29, 6	1, 12, 3, 7, 3	0.565
MDR-TB, no. (%)	1 (1.35%)	0 (0%)	1.000	2 (2.3%)	0 (0%)	1.000
**Comorbidity, no. (%)**						
Congestive heart failure	9 (12.2%)	5 (22.7%)	0.2996	9 (10.8%)	11 (42.3%)	<0.001
Chronic pulmonary disease	7 (9.46%)	6 (27.3%)	0.0685	18 (20.9%)	2 (7.7%)	0.152

Renal disease	2 (2.7%)	4 (18.2%)	0.024	2 (2.3%)	2 (7.7%)	0.230
Diabetes mellitus	9 (12.2%)	4 (18.2%)	0.487	16 (18.6%)	4 (15.4%)	1.000
Liver disease	2 (2.7%)	1 (4.6%)	0.546	1 (1.16%)	2 (7.7%)	0.134
Cerebrovascular disease	15 (20.3%)	7 (31.8%)	0.262	14 (16.3%)	6 (23.1%)	0.559
Neoplasm	5 (6.8%)	4 (18.2%)	0.204	3 (3.5%)	6 (23.1%)	0.005
Chronic corticosteroid treatment	4 (5.4%)	3 (13.6%)	0.195	3 (3.5%)	2 (7.7%)	0.329
**Clinical characteristics on admission**						
Body temperature, ℃	36.9±0.5	37.0±1.0	0.798	36.8±0.7	37.0±1.2	0.117
Heart rate, rate/min	83.0±15.1	86.4±16.9	0.567	85.3±15.9	88.4±15.6	0.341
Respiratory rate, breaths /min						
Respiratory failure (SaO_2 < 90%), no. (%)	10 (13.5%)	12 (54.6%)	<0.001	6(7.0%)	11(42.3%)	<0.001
Impaired consciousness, no. (%)	1 (1.35%)	4 (18.2%)	0.009	0 (0%)	4 (15.4%)	0.002
**Laboratory findings**						
BUN, (8.6-21.6 mg/dl) ***	18.4±13.3	21.5±12.4	0.238	16.1±7.7	22.7±15.6	0.078
TP, (6.7-8.1g/dl)	7.0±0.8	6.0±1.1	<0.001	7.4±0.8	6.1±0.9	<0.001
Alb, (3.9-4.9 mg/dl)	3.3±0.8	2.4±0.6	<0.001	3.2±0.8	2.3±0.5	<0.001
Cre, (0.70-1.17 mg/dl)	0.76±0.35	0.92±0.69	0.516	0.86±0.76	0.90±0.64	0.926
WBC, (3600-9200 /mm^3)	6655±2153	8100±4402	0.268	8076±3925	7569±3187	0.743
ESR, (2-10 mm/H)	63.6±32.7 (n=63)	68.1±37.3 (n=21)	0.545	46.5±28.2 (n=85)	37.8±34.3 (n=26)	0.076
CRP, (≦0.10 mg/dl)	4.0±3.8	9.3±5.5	<0.001	4.5±5.5	9.6±6.7	<0.001
SAA, (≦10 μg/ml)	333±372 (n=65)	541±466 (n=19)	0.065	345±537 (n=85)	615±783 (n=26)	0.007

Supplement Table 2. Correlation between the serum soluble CD206 and clinical parameters in patients with pulmonary tuberculousis

	Serum sCD206 Tenryu cohort		Serum sCD206 Shizuoka cohort	
	R	p	R	p
Age	0.367	0.003	0.240	0.013
Body mass index, Kg/m^2	-0.355	0.004	-0.267	0.005
P/F ratio, mmHg	-0.398	0.001	-0.588	<0.001
TP, g/dl	-0.547	<0.001	-0.480	<0.001
Alb, g/dl	-0557	<0.001	-0620	<0.001
BUN, mg/dl	0.179	0.161	0.242	0.012
Cre, mg/d	0.105	0.415	0.060	0.541
LDH, IU/l	0.430	<0.001	0.639	<0.001
CRP, mg/dl	0.457	<0.001	0.520	<0.001
SAA, mg/dl	0.332	<0.001	0.402	<0.001
Serum sCD206 ng/ml	0.684	<0.001	0.470	<0.001
WBC, /µl	0.032	0.804	0.108	0.267
HB, g/dl	-0.415	<0.001	-0.305	0.001
Plt, x 10^4/µl	-0.280	0.027	-0.289	0.003

P/F ratio, ratio of partial pressure arterial oxygen and fraction of inspired oxygen (PaO_2/FiO_2); TP, total protein; Alb, albumin; BUN, blood urea nitrogen; Cre, creatinine; LDH, lactate dehydrogenase; CRP, C-reactive protein; SAA, serum amyloid A; PCT, procalcitonin; WBC, white blood cell count; HB, hemoglobin; Ht, hematocrit; Plt, platelets.

Supplement Table 3. Characteristics of Pleural fluid with 100 patients

	TB pleurisy
No of patients	29
Sex, M/F	19 / 10
Age	79.5±11.7
TP (mg/dl)	4.3 ± 1.3
Glu (mg/dl)	118.2 ± 60.4
LDH (U/l)	484.9 ± 333.8
ADA (U/l)	84.3 ± 52.7

TB; tuberculosis, TP; total protein, Glu; glucose, LDH; lactate dehydrogenase, ADA; adenosine deaminase activity

Supplement Table 4. Diagnostic value of serum soluble CD206 predicts mortality of pulmonary tuberculous patients

Predictor	Sensitivity (%)	Specificity (%)	Likelihood	Sensitivity (%)	Specificity (%)	Likelihood	Sensitivity (%)	Specificity (%)	Likelihood
sCD206 (ng/ml)	Tenryu cohort			Shizuoka cohort			Combined cohort		
1,000	81.8	66.2	2.42	92.3	61.6	2.41	87.5	63.8	2.41
1,400	77.3	82.4	4.40	84.6	75.6	3.47	81.3	78.8	3.82
1,500	77.3	85.1	5.20	80.8	79.1	3.86	79.2	81.3	4.22
1,600	77.3	86.5	5.72	76.9	81.4	4.14	79.2	83.8	4.87
1,800	68.2	89.2	6.31	73.1	86.1	5.24	70.8	87.5	5.67
2,000	54.6	90.5	5.77	61.5	87.2	4.81	60.4	88.8	5.37

Supplement Table 5. The AUCs of predicting mortality in patients with pulmonary tuberculosis

sCD206 (ng/ml)	Tenryu cohort		Shizuoka cohort		Combined	
	AUC	95% CI	AUC	95% CI	AUC	95% CI
CD206, ng/ml	0.847	(0.756-0.939)	0.874	0.800-0.946	0.860	0.801-0.918
CRP, mg/dl	0.784	0.670-0.899	0.757	0.658-0.858	0.768	0.693-0.842
SAA, µg/ml	0.640	0.501-0.778	0.675	0.564-0.785	0.657	0.570-0.744
